# Supplementary material for: When the Optimal Is Not the Best: Parameter Estimation in Complex Biological Models
Source: PLoS One. 2010 Oct 25;5(10):e13283. doi: 10.1371/journal.pone.0013283 (PMC2963600; doi:10.1371/journal.pone.0013283)
Supplement: File S1 — Supplemental figures with the corresponding text. (0.70 MB PDF) [file pone.0013283.s001.pdf]

When the optimal is not the best: parameter estimation in complex biological models (Supplemental Material)

Diego Fernández Slezak [1,*], Cecilia Suárez [1], Guillermo A. Cecchi [2], Guillermo Marshall [1] and Gustavo Stolovitzky [2,*]
[1]Laboratorio de Sistemas Complejos, Depto. de Computación, FCEyN, UBA. Pabellón I, Ciudad Universitaria, (C1428EGA) Buenos Aires, Argentina
[2]Computational Biology Center, T.J.Watson Research Center, IBM. P.O. Box 218, Yorktown Heights, N.Y. 10598

September 20, 2010

1 Model Behavior

Although the model does not consider explicitly the saturation of the tumor size r_{bd} to a final stable radius, modifications of the parameter δ allow the model to reach saturation. This parameter sets the relationship between dead and living cell volume. So, with $\delta = 1$ both volumes are equal and the tumor will go on growing indefinitely. On the contrary, if $\delta = 0$ the dead cell is absorbed by the tumor, and eventually secreted, leaving a free space to be replaced by another nascent cell. In this case, when cell death compensates cell division, a saturation state can be achieved. The three possible regimes for the evolution of tumor radius as a function of time are illustrated in Fig. (S1). Different combinations of parameters lead to one of three possible evolutions: linearly increasing, saturating, and decreasing.

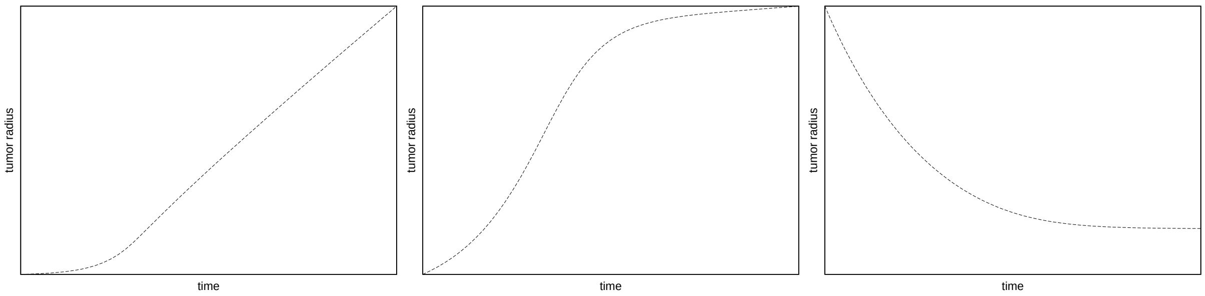

Figure S1: Avascular tumor growth model trends. Simulations were run using different parameter values, yielding three possible growth evolutions: (a) an exponential initial growth phase followed by indefinite linear growth, (b) an exponential initial growth phase followed by linear growth and followed by a saturation, and (c) a tumor of shrinking size.

*to whom correspondence should be addressed

2 Parameter Estimation

The parameters included in the tumor growth model are δ, σ, β, c_c, c_d and c_{bd}, which are represented by a 6-dimensional array θ, with components $(\delta, \sigma, \beta, c_c, c_d, c_{bd})$. In order to obtain numerical values for our model parameters, we minimize the differences between our tumor growth model and the data by finding the minima of the quadratic cost function [1]:

$$\chi^2(\theta) = \sum_{k=0}^{N} (y_k(\theta) - \text{data}_k)^2,$$

where $y_k(\theta)$ is the model's prediction for observation k which depends on the parameters θ, and data_k represents the experimentally measured data values (in our case, synthetic data) at observation k. In our particular application, synthetic experimental observations are taken at times t_k, and the value data_k corresponds to the radius of the spheroid at time t_k. The model prediction for the spheroid radius at time t_k is denoted by $y_k(\theta)$, and the sum is over all the time points t_k. For future reference, the residuals vector $\mathbf{f}$ is defined as $f_k = y_k(\theta) - \text{data}_k$.

The methods used for the minimization of the cost function are described below.

- **Levenberg-Marquardt** Introduced by Levenberg [2] and rediscovered by Marquardt [3], this method belongs to the optimization family called *descent methods* [1]. One of the most famous member of this family is the Gauss-Newton method [1], which calculates the descending direction using the Jacobian of the cost function. As the Gauss-Newton approach, this method uses the Jacobian of the cost function to find the descending direction, but incorporates a damping parameter μ. Let $\mathbf{J}$ be the Jacobian of the residuals $f_k(\theta) = y_k(\theta) - \text{data}_k$, i.e., $J_{ki} = \partial f_k / \partial \theta_i$ and $\mathbf{h}$ be the descending direction, then:

$$\begin{aligned} \textbf{Steepest descent:}\ & \mathbf{h}_{sd} = -\mathbf{J}^T \mathbf{f}, \\ \textbf{Gauss-Newton:}\ & (\mathbf{J}^T \mathbf{J}) \mathbf{h}_{gn} = -\mathbf{J}^T \mathbf{f}, \\ \textbf{LM:}\ & (\mathbf{J}^T \mathbf{J} + \mu \mathbf{I}) \mathbf{h}_{lm} = -\mathbf{J}^T \mathbf{f} \quad \text{with } \mu \geq 0. \end{aligned}$$

  With large values of μ, LM is close to the steepest descent method, and achieves a faster convergence if the current value of the parameters is far from the solution. On the other hand, for small values of μ, the method is close to the Gauss-Newton method, which is generally good in the final steps of the search. The strength of the LM method is that it may vary the value of μ to behave closer to the Gauss-Newton or the steepest descent methods. We followed Ref. [4] for the implementation of this method.

- **MIGRAD** This is another well known descent method included in the MINUIT package implemented by CERN [5]. It implements the Davidon-Fletcher-Powell (DFP) method, which belongs to a group of methods variously called quasi-Newton methods. Given a point θ_k in parameter space, the Newton-Raphson's method generates the search direction $\Delta\theta_k = H_k \mathbf{J_k}$ where H_k is the k-th update of the inverse of the Hessian matrix of $\chi^2(\theta)$, i.e. $H_{ij} = \frac{\partial^2 \chi^2}{\partial \theta_i \partial \theta_j}$, and $\mathbf{J}_k = \nabla \chi(\theta_k + \Delta\theta_k) - \nabla \chi(\theta_k)$. The quasi-Newton methods avoid the calculation of the Hessian directly and successively estimate the inverse of the Hessian matrix using only the gradients of the cost function. The update rule for the inverse Hessian in the DFP method is:

$$H_{k+1} = H_k + \Delta\theta_k \Delta\theta_k^T / \Delta\theta_k^T \mathbf{J}_k - (H_k \mathbf{J}_k)(H_k \mathbf{J}_k)^T / \mathbf{J}_k^T H_k \mathbf{J}_k.$$

- **Downhill Simplex** This is a direct search method for function minimization introduced in 1965 [6, 7]; we used its implementation as included in the MINUIT package. The Nelder-Mead method

is simplex-based, defined as the convex hull of $n+1$ vertices x_0, ..., x_n, e.g. a triangle in $\mathbb{R}^2$ or a tetrahedron in $\mathbb{R}^3$. From an initial starting simplex, the method performs a sequence of transformations of the working simplex S, decreasing the function values at its vertices computing one or more test points, together with their function values. This method does not compute the gradient or Hessian of the cost function.

- **Parallel Tempering** This is a physics-inspired method for space sampling and optimization introduced in [8]. Our implementation is based on [9]. In Parallel Tempering (PT) multiple independent replicas of a system are evolved, with each replica being at a different "temperature" than the others. For the thermodynamic analogy the energy function is taken to be the cost function. High temperature generally permits the sampling point to search a larger volume of the phase space while low temperature replicas search the local landscape. Neighboring simulations (i.e., adjacent temperatures) may exchange configurations, subject to the acceptance criterion (that depends on the temperature and cost function values of the configurations to be exchanged) given by the Metropolis algorithm. Note that the difference between parallel tempering and simulated annealing [10] is that the former allows the fast sampling of a big space whereas the latter tends to locally explore a smaller area of the same space. If the smallest of the temperatures of parallel tempering is sufficiently low, then parallel tempering can be considered both as a method for global search (for the replicas at higher temperatures) and local optimization (for the replicas at lower temperatures).

3 Growth Curves

In Fig. (S2) we show the growth curve corresponding to the minima found by PT and LM methods with the Gompertz curve used as surrogate experimental data. It is evident that all of these minima generate growth curves that are good matches to the experimental data, at least qualitatively.

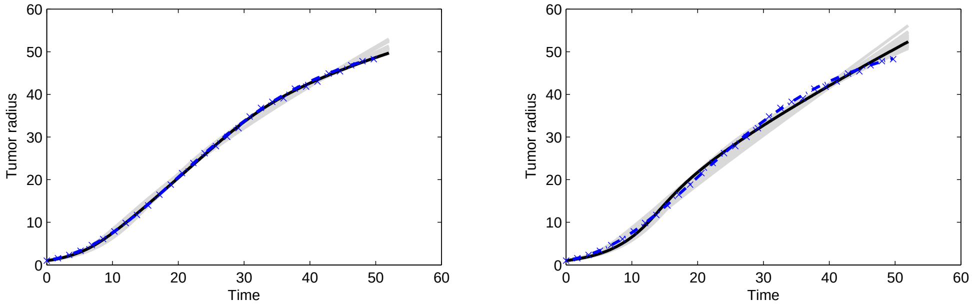

Figure S2: Growth curves for the best parameter values found by LM (left) and PT (right) are in gray color. The blue dashed line and x symbols show the Gompertz curve used to generate the experimental-like data and the solid black line shows the result of the model run using the optimal parameter set found according to each method.

4 Clustering

Fig. (S3) shows the clustering results for the 64 minima found by each compute node, for the LM (left panel) and PT (right panel) approaches.

In Figure S3 we divided the sets of minima into seven clusters for each method, and highlighted the identity of the clusters by red squares. It is clear that the clusters resulting from the LM optimization are tighter and more homogeneous within clusters than those resulting from PT. For each of the seven

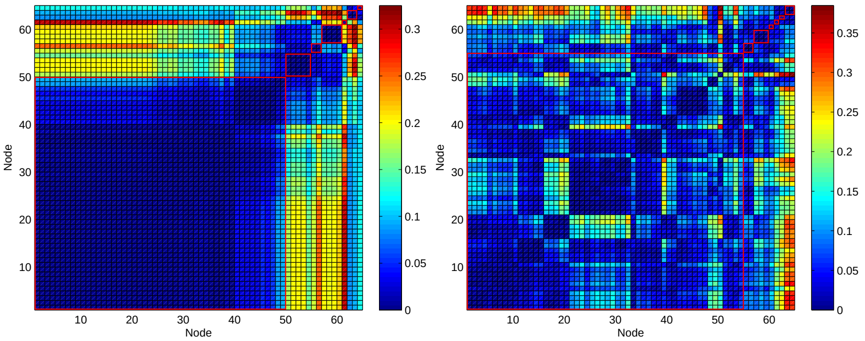

(a) Clustering for LM minima (b) Clustering for PT minima

Figure S3: Hierarchical clustering of the six-dimensional parameter vector obtained from the 64 minima found with LM and PT methods. Distances are calculated as one minus the sample correlation between points.

clusters and each method the mean value of each parameter was chosen as the representative solution. The resulting cluster centroids are listed in tables S1 and S2 for the LM and PT centroids respectively.

The resulting cluster centroids are listed in tables S1 and S2 for the LM and PT centroids respectively.

Table S1: Clustered minima values obtained by LM. Number of nodes included in each cluster are shown, as well as mean value and standard deviation of parameter set.

#	Parameter set for LM
2	$(1.0676 \times 10^{-1}, 7.4792 \times 10^{-1}, 9.0905 \times 10^{-2}, 2.1121 \times 10^{-1}, 1.2035 \times 10^{-8}, 9.0932 \times 10^{-1})$
49	$(7.8715 \times 10^{-2}, 9.7275^{-1}, 1.4764 \times 10^{-1}, 2.2737 \times 10^{-1}, 1.7400 \times 10^{-6}, 4.7007 \times 10^{-1})$<br>$\pm(2.0955 \times 10^{-2}, 2.1231 \times 10^{-2}, 1.0001 \times 10^{-2}, 5.3974 \times 10^{-2}, 9.3969 \times 10^{-6}, 9.9005 \times 10^{-2})$
2	$(3.1688 \times 10^{-1}, 7.8585 \times 10^{-1}, 1.1927 \times 10^{-1}, 5.5422 \times 10^{-2}, 1.3921 \times 10^{-8}, 4.3383 \times 10^{-1})$
1	$(2.1660 \times 10^{-1}, 6.5203 \times 10^{-1}, 1.4338 \times 10^{-1}, 5.0054 \times 10^{-2}, 1.0264 \times 10^{-10}, 9.0088 \times 10^{-1})$
4	$(1.9716 \times 10^{-1}, 9.9406 \times 10^{-1}, 1.4733 \times 10^{-1}, 6.9980 \times 10^{-1}, 5.3479 \times 10^{-3}, 9.6853 \times 10^{-1})$
1	$(3.0287 \times 10^{-1}, 7.5747 \times 10^{-1}, 1.5000 \times 10^{-1}, 6.2128 \times 10^{-2}, 2.1185 \times 10^{-9}, 5.8579 \times 10^{-1})$
5	$(4.7452 \times 10^{-2}, 8.8400 \times 10^{-1}, 1.4997 \times 10^{-1}, 4.0390 \times 10^{-1}, 2.1061 \times 10^{-7}, 9.6495 \times 10^{-1})$<br>$\pm(1.0187 \times 10^{-2}, 6.8654 \times 10^{-3}, 3.6469 \times 10^{-5}, 1.2923 \times 10^{-2}, 1.5646 \times 10^{-7}, 4.4001 \times 10^{-2})$

Table S2: Clustered minima values obtained by PT. Number of nodes included in each cluster are shown, as well as mean value and standard deviation of parameter set.

#	Parameter set for PT
54	$1.9098 \times 10^{-1}, 9.0086 \times 10^{-1}, 7.8273 \times 10^{-2}, 2.3818 \times 10^{-1}, 1.3364 \times 10^{-1}, 8.3954 \times 10^{-1}$<br>$\pm(6.6241 \times 10^{-2}, 7.1886 \times 10^{-2}, 3.5866 \times 10^{-2}, 1.4955 \times 10^{-1}, 1.2574 \times 10^{-1}, 1.1765 \times 10^{-1})$
1	$(3.7388 \times 10^{-1}, 7.3147 \times 10^{-1}, 1.0669 \times 10^{-1}, 6.5695 \times 10^{-2}, 5.7700 \times 10^{-4}, 4.8426 \times 10^{-1})$
2	$(4.7686 \times 10^{-1}, 8.8974 \times 10^{-1}, 1.3197 \times 10^{-1}, 1.0022 \times 10^{-1}, 1.8069 \times 10^{-3}, 4.1462 \times 10^{-1})$
3	$(3.6106 \times 10^{-1}, 8.9530 \times 10^{-1}, 1.0603 \times 10^{-1}, 6.5103 \times 10^{-2}, 2.0055 \times 10^{-1}, 6.8517 \times 10^{-1})$
2	$(3.2051 \times 10^{-1}, 9.1563 \times 10^{-1}, 1.2915 \times 10^{-1}, 2.0466 \times 10^{-1}, 3.9210 \times 10^{-2}, 7.3686 \times 10^{-1})$
1	$(3.3367 \times 10^{-1}, 7.2837 \times 10^{-1}, 1.2617 \times 10^{-1}, 6.6534 \times 10^{-2}, 8.7134 \times 10^{-3}, 6.6750 \times 10^{-1})$
1	$(4.1587 \times 10^{-1}, 9.9735 \times 10^{-1}, 1.0617 \times 10^{-1}, 5.1761 \times 10^{-2}, 1.0607 \times 10^{-1}, 4.6686 \times 10^{-1})$

5 Relative Error

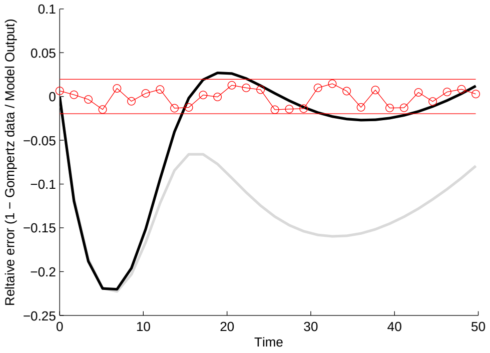

Figure S4: Relative error between Gompertz curve and model at best parameters. The red values show the relative error in the synthetic data. Red lines indicate two times the standard deviation of synthetic data. Black and gray lines correspond to the LM and PT minima, respectively.

References

[1] Björck A (1996) Numerical methods for least squares problems. Society for Industrial Mathematics.

[2] Levenberg K (1944) A method for the solution of certain nonlinear problems in least squares. Quart Appl Math 2: 164–168.

[3] Marquardt D (1963) An algorithm for least-squares estimation of nonlinear parameters. Journal of the Society for Industrial and Applied Mathematics 11: 431–441.

[4] Lourakis M (Jul. 2004). levmar: Levenberg-marquardt nonlinear least squares algorithms in C/C++. `http://www.ics.forth.gr/~lourakis/levmar/`. [Accessed on 3 Sep. 2009.].

[5] James F, Roos M (1975) Minuit-a system for function minimization and analysis of the parameter errors and correlations. Comput Phys Commun 10: 343.

[6] Nelder J, Mead R (1965) A Simplex Method for Function Minimization. The Computer Journal 7: 308–313.

[7] Singer A, Nelder J (2009) Nelder-mead algorithm. Scholarpedia 4: 2928.

[8] Marinari E, Parisi G (1992) Simulated tempering: a new Monte Carlo scheme. Europhys Lett 19: 451–458.

[9] Li Y, Mascagni M, Gorin A (2007) Decentralized Replica Exchange Parallel Tempering: An Efficient Implementation of Parallel Tempering Using MPI and SPRNG. Lecture Notes in Computer Science 4707: 507.

[10] Kirkpatrick S, Gelatt C, Vecchi M (1983) Optimization by Simulated Annealing. Science 220: 671–680.
